# Interpreting the Athlete’s ECG: Current State and Future Perspectives

**DOI:** 10.1007/s11936-018-0693-0

**Published:** 2018-11-19

**Authors:** Joyee Basu, Aneil Malhotra

**Affiliations:** Cardiology Clinical and Academic Group, St George’s, University of London, Cranmer Terrace, London, SW17 0RE UK

**Keywords:** Athlete, ECG, Consensus, Guidelines, Criteria, Screening

## Abstract

Sudden cardiac death (SCD) is the leading cause of death in athletes. A large proportion of these deaths are associated with undiagnosed cardiovascular disease. Screening for high-risk individuals enables early detection of pathology, as well as permitting lifestyle modification or therapeutic intervention.

ECG changes in athletes occur as a result of electrical and structural adaptations secondary to repeated bouts of exercise. Such changes are common and may overlap with patterns suggestive of underlying cardiovascular disease. Correct interpretation is therefore essential, in order to differentiate physiology from pathology. Erroneous interpretation may result in false reassurance or expensive investigations for further evaluation and unnecessary disqualification from competitive sports.

Interpretation of the athlete’s ECG has evolved over the past 12 years, beginning with the 2005 European Society of Cardiology (ESC) consensus, progressing to the ESC recommendations (2010), Seattle Criteria (2013) and the ‘refined’ criteria (2014). This evolution culminated in the recently published international recommendations for ECG interpretation in athletes (2017), which has led to a significant reduction in false positives and screening-associated costs. This review aims to describe the evolution of the current knowledge on ECG interpretation as well as future directions.

## Introduction

An athlete is defined as ‘one who participates in an organised team or individual sport requiring systematic training and regular competition against others, while placing a high premium on athletic excellence and achievement’ [1]. The sudden death of an athlete is a tragic event which is often highly publicised, particularly as athletes represent the healthiest individuals within society.

Sudden cardiac death (SCD) is the leading cause of death in athletes [2, 3] and a large proportion of these deaths are associated with undiagnosed cardiovascular disease [4, 5]. Young athletes demonstrate a 2.5-fold increased risk of SCD compared to sedentary individuals suggesting that sporting activity acts as a trigger for life-threatening arrhythmias in those with an underlying substrate [6]. The rate of SCD has previously been thought to be in the region of 1 in 50, 000 [2]. However, a recent study of 11, 000 adolescent football players identified an incidence of SCD of closer to 6.8 per 100,000 athletes [7].

Screening enables early detection of underlying cardiovascular disease, as well as enabling lifestyle modification and therapeutic intervention. A pre-participation screening programme comprising of history, physical examination and 12-lead ECG has been in practice in Italy for over 30 years [8]. This has led to an 89% reduction in SCD [9], primarily due to improved identification of cardiomyopathies through cessation of ongoing competitive physical activity. Most individuals at risk will not be identified from their history and examination alone, given that 80% are asymptomatic with SCD often the first presentation of underlying cardiovascular pathology [10]. On the contrary, ECG changes may be seen in up to 95% of patients with hypertrophic cardiomyopathy (HCM) and 80% of those with arrhythmogenic right ventricular cardiomyopathy (ARVC) [11]. Thus, use of the ECG improves the detection of early electrical changes which precede phenotypic expression. In addition, the ECG is essential to detect electrical conditions such as long QT syndrome, Brugada syndrome and Wolff-Parkinson-White (WPW) syndrome.

ECG adaptations in athletes are common and occur as a result of electrical and structural remodelling in response to repeated bouts of intense training regimes [12–18]. It is essential that an athlete’s ECG is correctly interpreted in order to differentiate physiological changes from pathology [19, 20]. Incorrect interpretation may result in an expensive diagnostic work up and unnecessary disqualification from competitive sports. This has significant psychological as well as financial implications for an affected individual. Conversely, if pathological changes are reported as normal, an athlete may be falsely reassured with potentially fatal consequences.

Interpretation of the athlete’s ECG has developed over time owing to new data and refinement of criteria based on validation in large and diverse cohorts. This has resulted in a significant reduction in false positives [21] and screening-associated costs [22]. This review aims to describe the evolution of current knowledge on ECG interpretation as well as highlight future directions in cardiac screening.

## ESC consensus statement (2005)

**Fig. 1:**
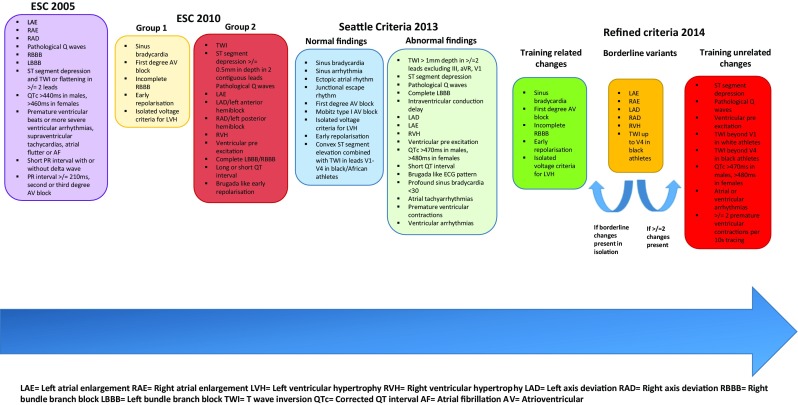
Evolution of the interpretation of the athlete’s ECG.

The first consensus statement regarding ECG interpretation in an athlete was compiled by the European Society of Cardiology (ESC) [23]. The group consisted of cardiovascular specialists and physicians from Europe. The criteria were predominantly based on individual expertise and small cohort experience and outlined the ECG phenotypes warranting further investigation (Fig. 1).

## Revised ESC recommendations (2010)

When applying the guidelines from the ESC consensus (2005), ECGs were found to be ‘abnormal’ in up to 50% of athletes [24] resulting in an unacceptably high false positive rate. The revised ESC criteria in 2010 divided ECG changes into common and training-related (group 1) and training-unrelated (group 2) changes (Fig. 1).

The group 1 changes have formed the basis for current ECG findings suggestive of normal athletic adaptation. Increased vagal tone in athletes can lead to sinus arrhythmia, first degree atrioventricular (AV) block (PR interval > 200 ms) and Mobitz type I AV block [25–27]. However, Mobitz type II and third degree AV block are rare in an athlete [28]. Incomplete right bundle branch block (RBBB) with a QRS duration < 120 ms occurs in 30–50% in athletes [29]. This is thought to be caused by enlargement of the right heart in response to training [29]. Early repolarisation is defined as elevation of the junction between the QRS-ST segments ≥ 0.1 mV [30] and is common in athletes. Although there have been studies suggesting a correlation between early repolarisation and ventricular fibrillation, there is still no conclusive evidence [31]. Voltage criterion for left ventricular hypertrophy (LVH) (S wave V1 + R wave V5/V6 = ≥ 35 mm) is common in athletes, and although it is also frequently observed on the ECGs of patients with HCM, less than 2% demonstrate LVH in isolation [32–34]. Voltage criterion for LVH in HCM is usually associated with other features such as *T*-wave inversion (TWI) in the inferior and/or lateral leads, Q waves and ST segment depression [35].

## The Seattle Criteria (2013)

Despite the ESC 2010 recommendations improving the specificity of the ECG to detect disease, the effect of ethnicity was unaccounted for. Black athletes are increasingly competing at every level of sport yet an alarmingly high number of false positive ECGs were being identified in this group [36]. The Seattle criteria were devised to help address this issue, by an international group of sports cardiologists who convened to update current criteria for ECG interpretation. Normal and abnormal ECG findings according to the Seattle criteria are summarised in Fig. 1. In addition to the common training-related findings originally listed in the ESC recommendations, the Seattle criteria also included ethnicity-specific ECG changes. Convex ST segment elevation combined with *T*-wave inversion (TWI) in leads V1-V4 in black athletes was classified as a normal ethnic variant. This was based on a study of 904 black athletes who were compared to white athletes, black controls and black HCM patients [37]. Anterior TWI (V1-V4) were present in 12.7% of black athletes with lower prevalence in black controls and HCM patients. In contrast to lateral TWI, anterior TWI was not associated with pathology.

The Seattle Criteria also redefined several existing abnormal ECG parameters (Table 1).

**Table 1:** Abnormal ECG parameters

ESC guidelines	Seattle criteria
TWI ≥ 2 mm in ≥ 2 adjacent leads(deep) or minor in ≥ 2 leads	TWI > 1 mm in depth in two or more leads V2-V6, II and aVF, or I and aVL (excluding III, aVR)
Pathological *Q* waves > 4 mm deep in any lead except II and aVR	Pathological Q waves > 3 mm in depth or > 40 ms in duration in two or more contiguous leads except III and aVR
Right axis deviation > 115°	Right axis deviation > 120°
Not defined	Profound sinus bradycardia < 30 bpm
PR interval < 120 ms with or without a delta wave	PR interval < 120 ms with a delta wave
Intraventricular conduction delay > 120 ms	Intraventricular conduction delay > 140 ms

Less conservative cut-offs for an abnormal QT interval were adopted by the Seattle criteria with an upper limit of 470 ms in males and 480 ms in females. This was based on a study of 2000 elite athletes, 0.4% of whom had a QT interval of 460–570 ms. In the absence of symptoms or family history, a QTc of < 500 ms was unlikely to represent pathology [38]. Moreover, the QT interval for short QT was reduced to less than 320 ms. This has been proposed as an arbitrary cut-off based on data from over 18,000 asymptomatic individuals [39]. The authors also recommended further investigation in those athletes with ≥ 2 premature ventricular ectopics (VEs) as multiple VEs are uncommon in athletes and present in < 1% [40].

## The ‘Refined Criteria’ (2014)

Subsequent to the publication of the Seattle criteria, evidence emerged that certain ECG changes assigned to the training-unrelated group, in isolation may not be representative of pathology. The ‘refined criteria’, [41] for the first time, included right (*p* wave > 2.5 mm in II, II and aVF), left atrial enlargement (*p* wave > 120 ms in I and II with the negative portion of the *p* wave ≥ 1 mm in depth and ≥ 40 ms in duration in V1), right and left axis deviation (− 30 to − 90°) and right ventricular hypertrophy (RVH) (*R* wave in V1 + S wave in V5 = ≥ 10.5 mm) in a borderline variant category. A large study of over 2000 athletes and nearly 10,000 controls identified that in those with isolated axis deviation or atrial enlargement there were no significant functional or structural abnormalities [42]. Moreover, similar to LVH, isolated RVH has not been shown to correlate with pathology [43]. The authors defined the presence of two or more borderline variants as abnormal, warranting further investigation (Fig. 1).

The effectiveness of the ‘refined’ criteria was also validated in a cohort of 1208 black athletes, 4297 white athletes and 103 athletes with HCM. The performance of the ‘refined’ criteria was compared to the ESC recommendations and Seattle criteria [41]. All three criteria identified 98.1% of those with HCM, indicating similar sensitivity in detecting cardiac pathology. The use of the ‘refined’ criteria reduced abnormal ECGs to 11.5% in black athletes and 5.3% in white athletes thus improving the specificity of the ESC recommendations from 40.3 to 82.4% in black athletes and 73.8 to 94.1% in white athletes.

## International recommendations for electrocardiographic interpretation in athletes

**Fig. 2:**
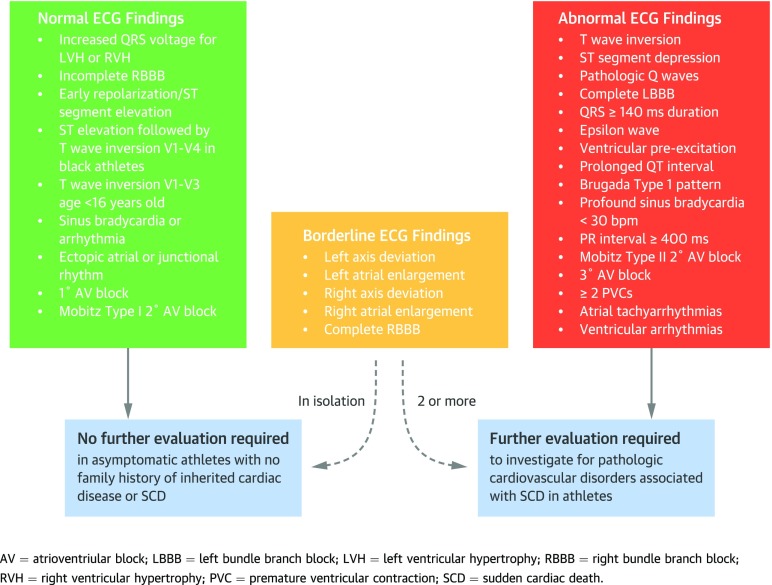
International recommendations for ECG interpretation in athletes.

The evolution of the interpretation of the athlete’s ECG has culminated in the recent publication of the international recommendations for ECG interpretation in athletes [44] (Fig. 2).

Building on previous criteria, the international recommendations also included the juvenile ECG pattern, up to the age of 16 years, as a normal ECG finding [45, 46]. The authors also reclassified TWI in V1-V4 with preceding convex ST elevation in black athletes as a normal finding.

The international recommendations redefined abnormal TWI to be that which extended beyond V2. This was based on a large study of 14,000 Caucasians which demonstrated that anterior TWI had a prevalence of 2.3%, was more common in females and athletes and was confined to V1 and V2 in most. TWI only extended beyond V2 in 1% of females and 0.2% of males [47]. Other additions to abnormal ECG findings included a PR interval of > 400 ms, and only the type I Brugada pattern was considered to be diagnostic (coved RSR pattern which has ST segment elevation ≥ 2 mm and inversion of the terminal portion of the *T* wave in V1, V2 and V3) [48].

RBBB was added to the borderline category, although the evidence for whether RBBB is pathological in the long term is lacking [49]. A single study of 510 US athletes showed that athletes with RBBB had larger LV dimensions, a reduced ejection fraction but the fractional area change was preserved. They did not display any evidence of pathology [50]. Unlike RBBB, left bundle branch block (LBBB) is found in < 1000 athletes [35, 49] and therefore should be considered pathological until proven otherwise. Finally, *Q* waves were redefined as a *Q*/*R* ratio of ≥ 0.25 or ≥ 40 ms in two or more contiguous leads (except III and aVR).

## Discussion

The evolution of ECG guidelines has enhanced identification of cardiovascular pathology and dramatically reduced false positives. This has not only benefitted specialists but also physicians with limited experience within the field of sports cardiology [51, 52]. Revision of the criteria has also resulted in significant cost savings [53]. A recent paper [22] compared the costs associated with the use of the ESC recommendations, Seattle criteria and ‘refined’ criteria. The number of abnormal ECGs, most of which were false positives reduced from 11.2 to 6% with the Seattle criteria and 4.3% with the ‘refined’ criteria. The reduction in the number of false positives was associated with a 20% cost reduction of screening, from $110 per athlete with the ESC to $92 and $87 per athlete, with the Seattle and ‘refined’ criteria, respectively.

Although there have been considerable advances in the interpretation of the athlete’s ECG, there remain areas which require further clarification. The significance of indices such as biphasic TWI, isolated inferior TWI and RBBB in athletes is not well understood, [49] and further investigation of the athlete is currently advised. It also remains unclear how to differentiate a black athlete’s ECG from arrhythmogenic cardiomyopathy as both are characterised by anterior TWI. A recent study by Calore et al. attempted to identify novel ECG markers of pathology to resolve this issue. The authors showed that in athletes anterior J point elevation ≥ 1 mm preceding TWI in leads V1-V4 excluded cardiomyopathy with 100% negative predictive value [54], and suggested that anterior TWI associated with minimal or absent J point elevation is suggestive of cardiomyopathy. However, more recent studies have challenged that claim, as J-point elevation may be absent in 30% of male and 70% of female athletes. [48]

Consideration should also be given to the growing population of mixed race athletes and veteran amateur athletes [55]. Mixed race athletes have been poorly represented in existing studies and although black ethnicity is reported as a single group, recent data suggest that this is far from the truth, with black athletes of west and central African descent the ones more likely to exhibit ECG anomalies. [56] Finally, long-term follow up studies of athletes are required in order to identify false negative rates, given that the phenotypic manifestations of certain conditions may occur later in life.

## Conclusion

Interpretation of the ECG has undergone significant modifications over the last two decades. As a result, the false positive rates and associated costs have dramatically declined. There appears to be scope to further refinement of the ECG criteria in order to improve sensitivity and specificity. One must however, pay heed to limitations of the ECG which may not demonstrate certain pathologies such as premature coronary disease, aortopathies and anomalous coronary arteries. Moreover, ECGs in patients with underlying pathology may be normal. Therefore, although the guidelines serve as an important reference for clinicians, the ECG should be interpreted in the context of symptoms, family history and personal experience. Where possible, assessment should be performed by a specialist with knowledge and experience of inherited cardiac conditions and sports cardiology.
